# 4-Ethylphenyl-Cobalamin Impairs Tissue Uptake of Vitamin B_12_ and Causes Vitamin B_12_ Deficiency in Mice

**DOI:** 10.1371/journal.pone.0075312

**Published:** 2013-09-20

**Authors:** Elena Mutti, Markus Ruetz, Henrik Birn, Bernhard Kräutler, Ebba Nexo

**Affiliations:** 1 Department of Clinical Biochemistry, Aarhus University Hospital, Aarhus, Denmark; 2 Institute of Organic Chemistry and Centre of Molecular Biosciences, University of Innsbruck, Innsbruck, Austria; 3 Department of Nephrology, Aarhus University Hospital and Department of Biomedicine, Aarhus University, Aarhus, Denmark; Bauer Research Foundation, United States of America; University of Melbourne, Australia

## Abstract

Co_β_-4-ethylphenyl-cob(III) alamin (EtPhCbl) is an organometallic analogue of vitamin B_12_ (CNCbl) which binds to transcobalamin (TC), a plasma protein that facilitates the cellular uptake of cobalamin (Cbl). In vitro assays with key enzymes do not convert EtPhCbl to the active coenzyme forms of Cbl suggesting that administration of EtPhCbl may cause cellular Cbl deficiency. Here, we investigate the *in vivo* effect of EtPhCbl in mice and its ability, if any, to induce Cbl deficiency. We show that EtPhCbl binds to mouse TC and we examined mice that received 3.5 nmol/24h EtPhCbl (n=6), 3.5 nmol/24h CNCbl (n=7) or NaCl (control group) (n=5) through osmotic mini-pumps for four weeks. We analyzed plasma, urine, liver, spleen, submaxillary glands and spinal cord for Cbl and markers of Cbl deficiency including methylmalonic acid (MMA) and homocysteine (tHcy). Plasma MMA (mean±SEM) was elevated in animals treated with EtPhCbl (1.01±0.12 µmol/L) compared to controls (0.30±0.02 µmol/L) and CNCbl (0.29±0.01 µmol/L) treated animals. The same pattern was observed for tHcy. Plasma total Cbl concentration was higher in animals treated with EtPhCbl (128.82±1.87 nmol/L) than in CNCbl treated animals (87.64±0.93 nmol/L). However, the organ levels of total Cbl were significantly lower in animals treated with EtPhCbl compared to CNCbl treated animals or controls, notably in the liver (157.07±8.56 pmol/g vs. 603.85±20.02 pmol/g, and 443.09±12.32 pmol/g, respectively). Differences between the three groups was analysed using one-way ANOVA and, Bonferroni post-hoc test. EtPhCbl was present in all tissues, except the spinal cord, accounting for 35-90% of total Cbl. In conclusion, treatment with EtPhCbl induces biochemical evidence of Cbl deficiency. This may in part be caused by a compromised tissue accumulation of Cbl.

## Introduction

**Figure 1:**
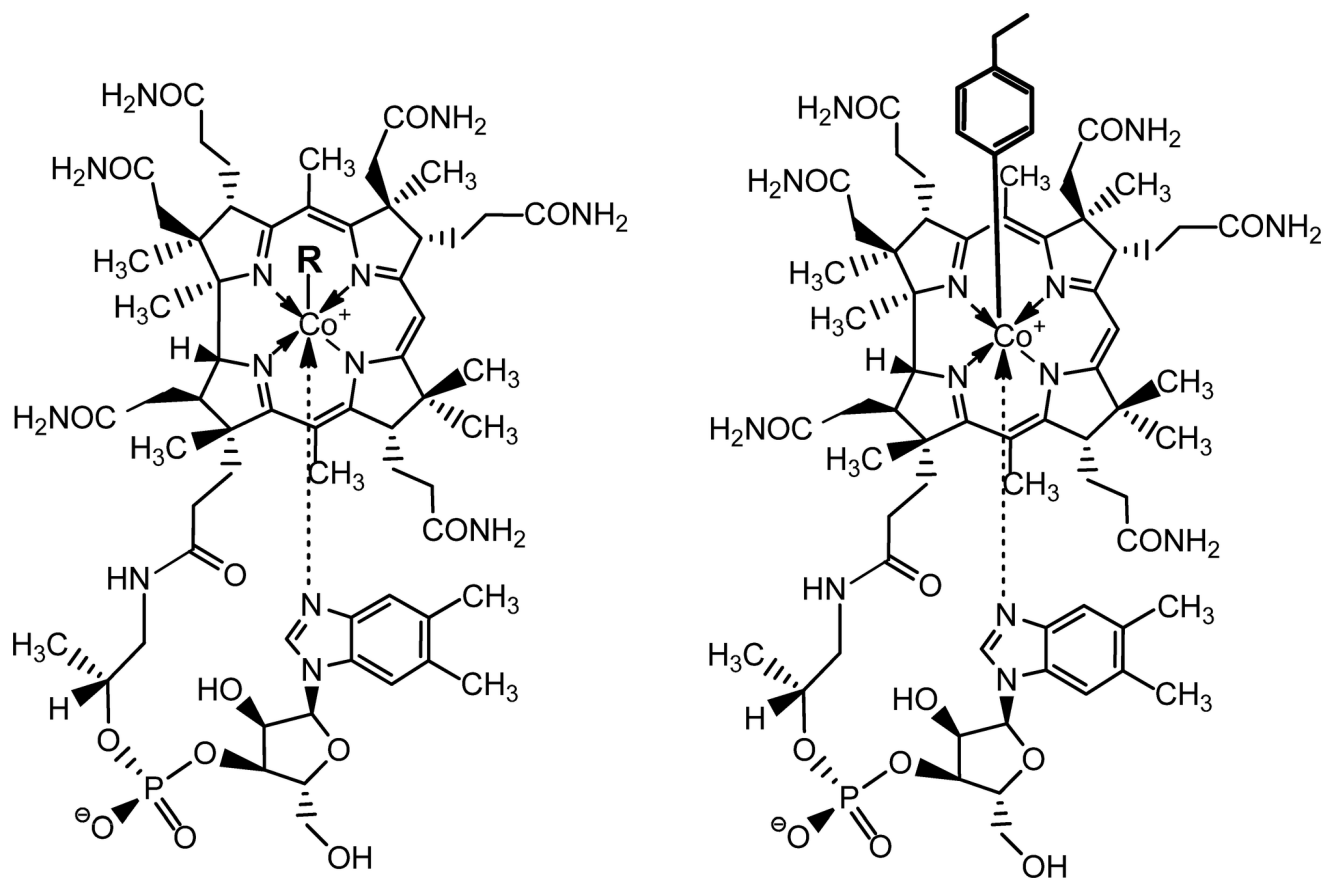
Structural formulas of some relevant cobalamins. Left: Active forms of cobalamins. R denotes any of the following ligands: 5’-deoxyadenosyl (5′-deoxyadenosyl-cobalamin (AdoCbl)); methyl (methylcobalamin (MeCbl)); CN (vitamin B_12_ (CNCbl)) or H_2_O (aquocobalamin (H_2_OCbl)). Right: Structural formula of Co_β_-4-ethylphenyl-cob(III) alamin (EtPhCbl) [3].

Cobalamins (Cbls) are delivered in several molecular forms differing by the axial ligand attached to the cobalt atom bound by the corrin macro ring (Figure 1). In oral vitamin supplementation, this ligand is a cyanide (vitamin B_12_, CNCbl) or a hydroxy (aquocobalamin, H_2_OCbl) group. Within the cell, the various forms of Cbl are reduced by the enzyme methylmalonic aciduria and homocystinuria type C protein (CblC), and, subsequently, 5′-deoxyadenosyl-cobalamin (AdoCbl) and methylcobalamin (CH _3_Cbl) are formed. These are coenzymes for the two known mammalian Cbl dependent enzymes. L-methylmalonyl-Coenzyme A (CoA) mutase [EC 5.4.99.2] catalyzes the conversion of L-methylmalonyl-CoA to succinyl-CoA, while methionine synthase [EC 2.1.1.13] catalyzes the conversion of homocysteine (tHcy) to methionine, which is linked to the simultaneous conversion of N^5^-methyltetrahydrofolate to tetrahydrofolate [1,2]. A Cbl analogue not reduced by CblC is expected to inhibit Cbl metabolism resulting in the symptoms and findings of Cbl deficiency. A mild, chronic deficiency of Cbl, caused by an impaired uptake of the vitamin, is quite common in the elderly population. Severe Cbl deficiency may induce megaloblastic anemia and a neuropathy known as subacute combined degeneration and, characterized by irregular demyelination of the white matter and astrogliosis [3].

Recently, we reported on such a metabolically inert Cbl form, Co_β_-4-ethylphenyl-cob(III) alamin (EtPhCbl) [4]. The upper ligand of EtPhCbl is resistant to cleavage by CblC, and, therefore, EtPhCbl cannot be converted into the active Cbl coenzyme forms. EtPhCbl binds to the human Cbl transporters, intrinsic factor and transcobalamin (TC) [4]. Thus, we hypothesize that large amounts of EtPhCbl are transported into cells in competition with endogenous Cbl and, when internalized, EtPhCbl inhibits Cbl metabolism leading to a Cbl-deficient state.

Here, we explore the *in vivo* effects of four weeks treatment with EtPhCbl using a mouse model. We report that EtPhCbl is able to induce a Cbl deficiency and at the same time impair the cellular internalization of Cbl.

## Materials and Methods

### Chemicals

Crystalline EtPhCbl was prepared by the authors as described [4]. In darkness, aqueous solutions of EtPhCbl are stable at neutral pH (pH 7) and low pH (pH 2) at room temperature or higher temperatures (100 °C), but such solutions of EtPhCbl are converted to H_2_OCbl upon exposure to bright daylight [4].

TC was derived from extracts of mouse submaxillary gland [5].

### EtPhCbl Binding to mouse TC

**Figure 2:**
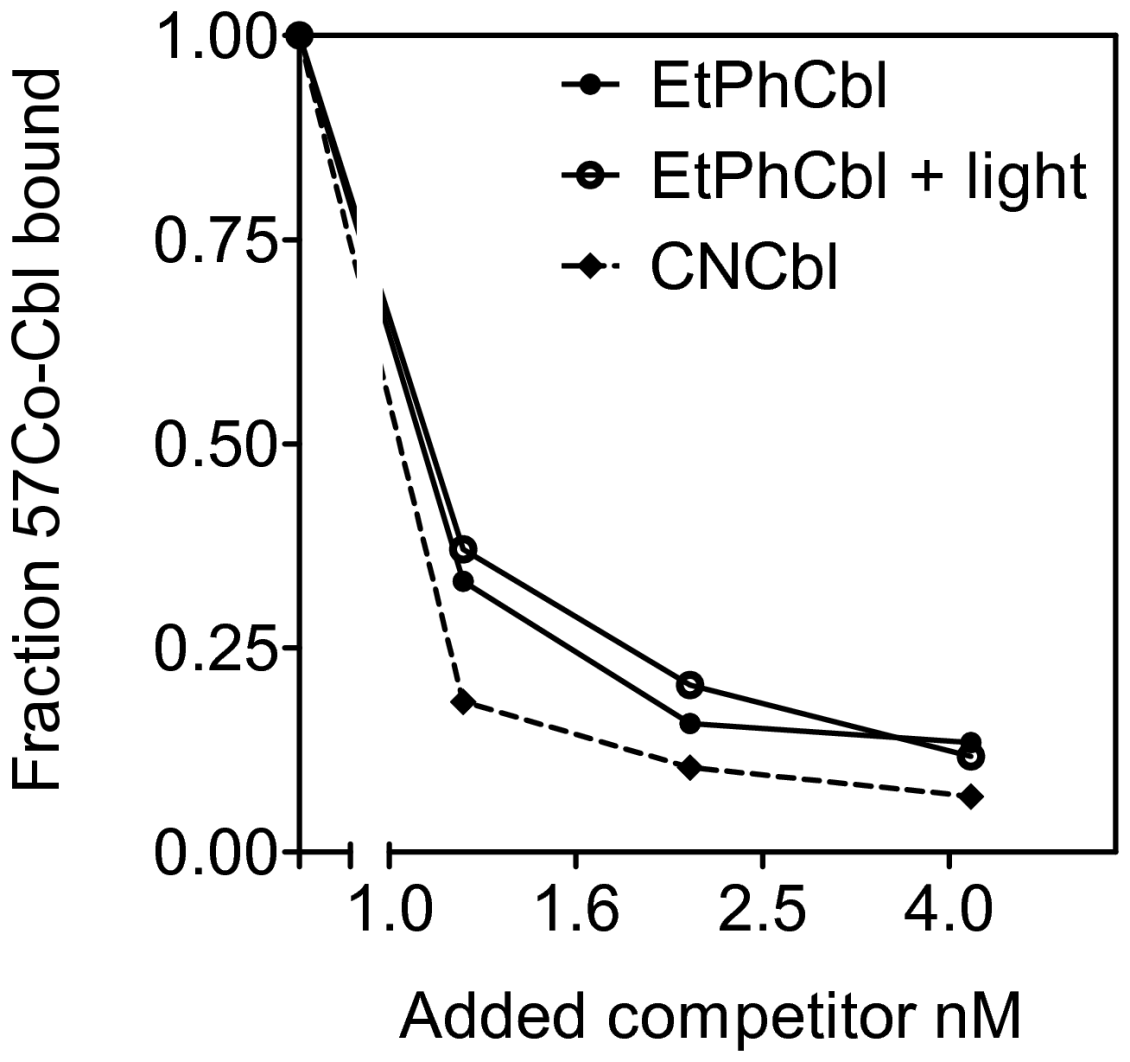
Binding of Co_β_-4-ethylphenyl-cob(III) alamin (EtPhCbl) to mouse TC. Increasing concentration of EtPhCbl (with and without exposure to light) or vitamin B_12_ (CNCbl) was incubated with^57^[Co]-Cbl and mouse TC. The quantity of bound^57^[Co]-Cbl was expressed relative to the amount bound when only^57^[Co]-Cbl was present. A logarithmic scale was used on the X axis.

The ability of the EtPhCbl to bind mouse TC was analyzed using a competitive assay [6] (Figure 2). In brief, EtPhCbl (10 nM dissolved in 0.1% phosphate buffered albumin (PBA) (0.1M phosphate 0.1% bovine albumin (Sigma, Brøndby, Denmark), pH 8.0) was divided into two portions. One was exposed to daylight for 24 hrs before the experiment in order to convert EtPhCbl to H_2_OCbl. The samples were diluted with PBA to final concentrations ranging from 0 nM to 4.2 nM, and mixed with^57^[Co]-Cbl (MP Biomedical, Santa Ana, CA) (0.06 nM) tracer solution prior to addition of the mouse TC (0.6 nM). The mixture was incubated for 18 hours at 4°C. TC bound Cbl was harvested in the supernatant after precipitation of free Cbl with hemoglobin coated charcoal as previously described [6]. Radioactivity was measured in a Wizard Automatic Gamma Counter (PerkinElmer, Waltham, MA). For comparison, CNCbl (Sigma) diluted to the same concentrations as EtPhCbl was also examined.

Due to light sensibility of the EtPhCbl conjugates, the experiment was carried out in dim light unless otherwise indicated.

### Animal studies

All animal experiments were carried out in accord with the animal care license provided and approved by the Danish National Animal Experiments Inspectorate (provision no 2010/561-1855). Eighteen, age-matched (7 weeks of age), female mice (strain 129.S6) were divided in three groups: (i) control mice (n = 5), (ii) CNCbl-loaded mice (n = 7), and (iii) EtPhCbl-loaded mice (n = 6). The surgical procedures employed were essentially as previously described [7]. In brief, the mice were anesthetized with isoflurane (IsoFlo® Vet, Abbott Laboratories, Abbott Park, IL), and osmotic minipumps (Mini-Osmotic Pump Model 2004, Alzet, Cupertino, CA) were inserted subcutaneously following the manufacture’s instruction. Prior to insertion, the pumps were filled with either (i) 200 µL 0.9% NaCl (control mice), (ii) 0.58 mM CNCbl in 200 µL 0.9% NaCl (CNCbl mice), or (iii) 0.58 mM EtPhCbl in 200µL 0.9% NaCl (EtPhCbl mice) and equilibrated overnight. The mice were housed in individual cages for three days after surgery with analgesics administered to the drinking water (buprenorphine hydrochloride 0.06 mg/ml). All mice were fed on a standard mouse chow (Altromin maintenance diet for rats and mice (1324) (19 pmol/g Cbl Altromin, Lage, Germany)) with free access to food and water. The mice were weighed and their wellbeing was observed just after insertion of the pumps (day 0) and on days 5, 12, 19, and 27. The mice were sacrificed after four weeks of treatment using minipumps.

### Urine and blood collection

Twenty-four hour urine samples were collected. The mice were placed in metabolic cages with free access to water on the day before insertion of the pumps (day -1) and on days 5, 12, 19, and 27. Prior to urine collection, each mouse received an intraperitoneal injection of 250 µL 0.9% NaCl to increase urine output. One µL of 20% Na-azide was added to each urine collection tube to prevent bacterial growth.

On the day of sacrifice (day 28), mice were anaesthetized with isoflurane, and blood was collected from the inferior caval vein. One aliquot (~200 µL) of blood from each mouse was transferred to a dry EDTA tube for hematological analysis. The remaining blood was collected in heparinized tubes, and plasma was prepared by centrifugation at 4,000 G for 8 minutes at room temperature. Urine and plasma were stored at −20 °C until analyzed.

Due to light sensibility of the EtPhCbl conjugates, the blood collection and further handling of samples from EtPhCbl mice were carried out in dim light.

### Organ collection

Immediately after the mice were sacrificed on day 28, liver, kidney, spleen, submaxillary glands and spinal cord were collected and snap-frozen in liquid nitrogen. The organs were stored at −80 °C until further processing. Due to light sensibility of the EtPhCbl conjugates, the collection and further handling of samples from EtPhCbl mice were carried out in dim light.

### Crude tissue extraction

Crude extracts of aliquots of tissue were prepared in homogenization buffer (10 mM PIPES pH 7.4 (Sigma), 1 mM EGTA (Sigma), 3 mM MgCl_2_, 6H_2_O (Merck, Damstadt, Germany), 400 mM NaCl, 2 tablets per 25 mL buffer of proteinase inhibitor cocktail, Cat. No. 11697498001, Roche Diagnostics, Mannheim, Germany) as described previously [5]. Briefly, the tissue was homogenized using a tissue ruptor (Qiagen, Copenhagen, Denmark) and sonicated (MSE probe universal) 3 times 10 seconds. The sample was centrifuged for 40 minutes at 20000 G at 4 °C. The supernatant was kept at -20 °C until analyzed. The tissue-to-buffer ratios were: 231g/L (kidney); 400g/L (liver), 40 g/L (spinal cord), 40 g/L (spleen), and 140g/L (submaxillary glands).

### Biochemical analysis

Hemoglobin concentration was determined within two hours after collection using EDTA blood on a Sysmex XE-2100 Automated Hematology System (Sysmex Corporation).

Fifty µL of heparinized plasma from each mouse was sent to BeVital (http://www.bevital.no/) for analysis of methylmalonic acid (MMA), tHcy, total cysteine, and methionine levels using standardized GC-MS methods.

Cbl levels were measured in urine, plasma, and the supernatant from the crude protein extract (liver, kidney, submaxillary gland, spleen, spinal cord) using a competitive electrochemiluminescence immunoassay on a Cobas 6000e immunoassay system and the analytical kit supplied by the manufacturer (Roche Diagnostics). When required the samples were diluted in 0.9% NaCl prior to analysis. The samples from EtPhCbl mice were exposed to daylight for 24 hrs before analysis.

Cbl level in urine samples was normalized to the concentration of creatinine. Creatinine levels were measured employing an enzymatic assay on a Cobas c311 system using the analytical kit supplied by the manufacturer (Roche Diagnostics).

MMA in urine of EtPhCbl mice was measured using LC-MSMS (1290 LC-system and 6490 MS-detector; Agilent) [8] and normalized to the concentration of creatinine.

The different forms of Cbl present in organs and plasma of EtPhCbl mice were determined by HPLC separation followed by measurement of Cbl in the eluted fractions. Pools of plasma, kidney, liver, spinal cord, spleen, or submaxillary gland were prepared by mixing equal amounts of plasma or tissue extracts from all the mice in the EtPhCbl group. Cbl was extracted from each sample by acidic denaturation and boiling in the presence of KCN [9].

The HPLC apparatus (HP 1100, Agilent Technologies) was fitted with a precolumn (SecurityGuard Cartridge, Phenomenex) followed by a reversed-phase column (Luna 3u reversed-phase C18 150 mm × 4.6 mm, Phenomenex), which was maintained at 20 °C. A gradient of acetonitrile in 10 mmol/L phosphoric acid, pH 5, increasing from 2% to 40% in 20 minutes, and, subsequently, from 40% to 60% in 4 minutes was applied 4 minutes after injection of 90 µL of samples, with a flow rate of 1.0 mL/min. Post column fractions were collected for every 60 seconds between 4 and 25 minutes after injection, and the eluent in the fractions was lyophilized (Heto-Vac, Denmark). The Cbl concentration in the post column fractions was determined using an ELISA-based method employing human TC [10], and the total amounts of Cbl in each fraction were calculated and used to measure the peak areas in the resulting chromatograms. Standard solutions containing 400 pmol/l was employed to determine the retention times for H_2_OCbl (7 min), CNCbl (10 min), AdoCbl (11 min), CH _3_Cbl (13 min) (all from Sigma) and EtPhCbl (16 min). The EtPhCbl solutions collected prior to the experiment as well as from the minipump at the end of the experiment were also analyzed. The experiment was carried out in dim light until the Cbl forms had been separated by HPLC.

### Statistical analysis

**Figure 3:**
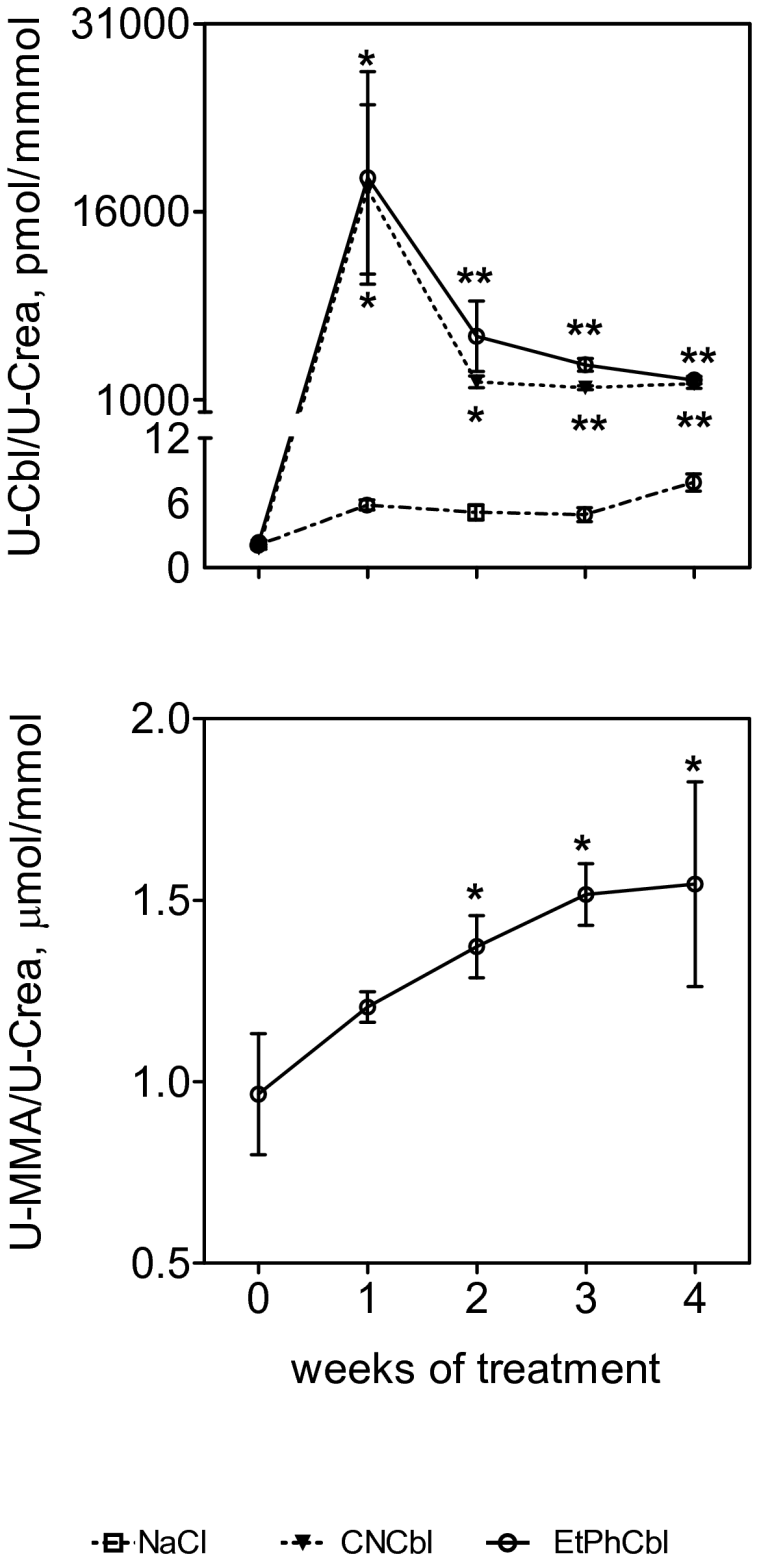
Cbl and MMA levels in mouse urine. Cbl (upper panel) and MMA (lower panel) levels in urine collected before and one to four weeks after insertion of minipumps delivering NaCl (controls, n=5) or 3.5 nmol/day vitamin B_12_ (CNCbl, n=7) or Co_β_-4-ethylphenyl-cob(III) alamin (EtPhCbl, n=6). Mean±SEM of Cbl/creatinine (Cbl) levels are shown for all three groups, and mean±SEM of MMA/creatinine levels are shown for mice treated with EtPhCbl. For both Cbl and MMA level, unpaired t-test, Welch correction, was employed to compare the difference between each experimental group at each time point and the baseline values (time 0). ^*^ p<0,05; ^**^p<0.0001 vs. baseline values of each experimental group. Note that the Y-scale for Cbl is presented in two segments. crea: creatinine; u: urinary.

**Figure 4:**
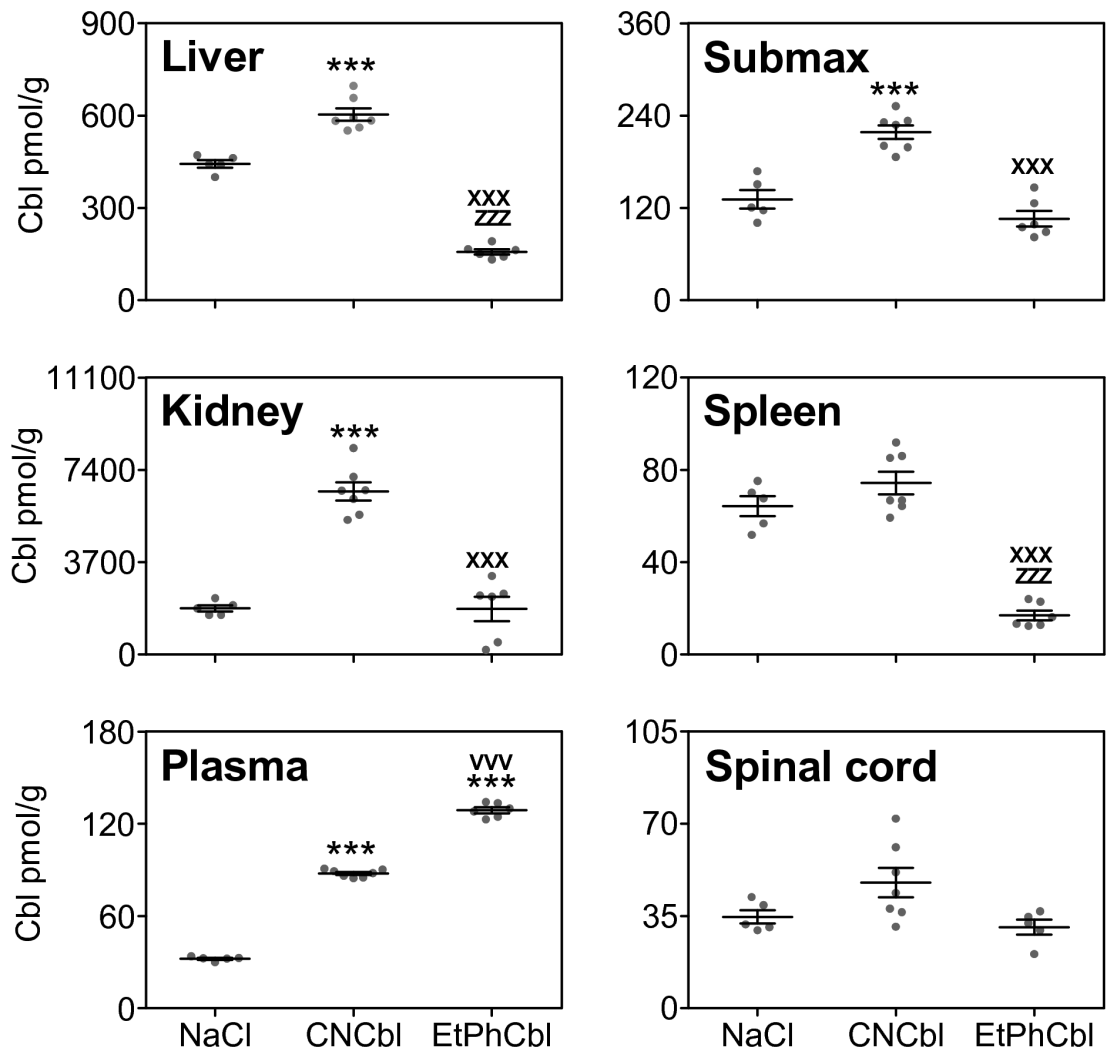
Cbl content in plasma and tissue extracts from mice. Scattergram of Cbl levels in plasma and tissues of mice treated for four weeks with NaCl (controls, n=5), 3.5 nmol/day vitamin B_12_ (CNCbl, n=7) or Co_β_-4-ethylphenyl-cob(III) alamin (EtPhCbl, n=6). The horizontal lines represent the mean level ± SEM. The figure shows the content of Cbl expressed per gram wet weight of tissue or per ml (plasma). One way ANOVA and Bonferroni post-hoc test were employed to compare Cbl levels between the groups. For all the groups ANOVA gave an F value which is statistically significant with three degrees of freedom. The levels of statistical significance of Bonferroni test are: *** indicates increased values, p< 0.001 compared to controls, zzz indicates decreased values, p<0.001 compared to controls, xxx indicates decreased values, p<0.001 compared to CNCbl, VVV indicates increased values, p<0.001 compared to CNCbl.

In Figure 3, the differences between each experimental group at each time point and the baseline value (time 0) were compared using unpaired t-test, Welch correction. In Table 1 and Figure 4, for each molecule in each tissue the homoscedasticity of the variances of the three groups of animals (NaCl, CNCbl and EtPhCbl mice) was tested using Bartlett’s test showing no statistically significant differences between variances. Differences in the means for the different groups was tested using one-way ANOVA followed by Bonferroni post hoc. The levels of statistical significance are shown in Table 1 and Figure 4. A P-value of 0.05 or less was considered statistical significant in all tests.

**Table 1:** Body weight and Cbl-related parameters in blood removed from mice treated for 27 days with continuous delivery of NaCl through osmotic minipumps (control), 3.5 nmol/day CNCbl or 3.5 nmol/day EtPhCbl.

	**Control (n=5)**	**CNCbl (n=7)**	**EtPhCbl (n=6)**
Body weight (g)	18.99±0.47	19.52±0.33	19.30±0.34
*Haematological parameters in blood*			
Hemoglobin concentrations (mmol/L)	9.70±0.10	9.10±0.10	9.00±0.10^*^
*Cbl-related biochemical parameters in plasma*			
Cbl (nmol/L)	32.23±0.63	87.64±0.93**	128.82±1.87**,††
MMA (µmol/L)	0.30±0.02	0.29±0.01	1.01±0.12**,††
tHcy (µmol/L)	7.89±0.56	6.64±0.30	11.63±0.33**,††
Cysteine (µmol/L)	197.91±11.65	200.92±6.66	85.00±5.76
Methionine (µmol/L)	70.66±2.01	83.46±2.91*	72.75±3.52

* p<0.05 vs: Control; **p<0.001 vs: Control, ††p< 0.001 vs. CNCbl

Mean± SEM is indicated. One way ANOVA and Bonferroni post-hoc test were employed to compare levels between the groups. For all the parameters, except body weight and cysteine, ANOVA gave an F value which is statistically significant with three degrees of freedom. The levels of statistical significance of Bonferroni test are:

## Results

### EtPhCbl binds to mouse TC

EtPhCbl is characterized by its light sensitive upper ligand (Figure 1) and is converted to H_2_OCbl upon exposure to light [4]. We explored the binding of EtPhCbl to mouse TC and report binding characteristics identical to those of H_2_OCbl (EtPhCbl converted to its aquoform by exposure to light) and comparable to the binding characteristics of CNCbl (Figure 2). We conclude that mouse TC - like human TC [4] - recognizes EtPhCbl as a base-on Cbl.

### Urine collection, biochemical analysis and body mass data

Using our previously established mouse model for administration of Cbl through osmotic mini-pumps [7], we analyzed the effects of four weeks of continuous treatment with EtPhCbl compared to treatment with NaCl or CNCbl. HPLC analysis of the EtPhCbl solution collected at the beginning and, from the minipumps, after the treatment period revealed a single peak eluting as EtPhCbl, hence confirming that EtPhCbl was intact throughout the study (data not shown).

The urinary excretion of Cbl was similar in animals treated with CNCbl and EtPhCbl. In both groups the excretion was more than thousand fold higher than in the controls consistent with an efficient delivery of the analogue (Figure 3). As expected, we found high levels of plasma Cbl in mice treated with CNCbl. Surprisingly, plasma Cbl levels were significantly higher in mice treated with EtPhCbl than in mice treated with CNCbl (Table 1, Figure 4).

The mice treated with EtPhCbl showed an increase in urinary MMA excretion after one week of treatment, and after two weeks of treatment, the level was significantly higher than in the control mice (Figure 3).

After four weeks of treatment, plasma markers of Cbl deficiency (MMA and tHcy) were increased in mice treated with EtPhCbl while unchanged or decreased in mice treated with CNCbl (Table 1). As previously reported [7], treatment with CNCbl resulted in an increased level of methionine and no change in cystein (Table 1). Blood hemoglobin concentrations were similar in the two treated groups, but; slightly lower than the level in the control mice (Table 1). We observed no difference in the body weight (Table 1) or any clinical sign of toxicity or Cbl deficiency (data not shown).

### Tissue levels of Cbl and EtPhCbl

Following four weeks of treatment with CNCbl, we observed the expected increase in tissue Cbl, levels, although significant only for kidney, liver and submaxillary gland (Figure 4). In contrast, mice treated with EtPhCbl revealed Cbl levels in liver and spleen approximately one third of the level in control animals and fourfold lower than the level in animals treated with CNCbl (157.07±8.56 pmol/g vs. 443.09±12.32 pmol/g and 603.85±20.02 pmol/g, respectively, in liver, and 16.90±2.13 pmol/g vs. 64.30±4.35 pmol/g and 74.32±4.87 pmol/g in spleen). All other organs studied also showed considerably lower Cbl values in animals treated with EtPhCbl compared to the group treated with CNCbl (Figure 4).

### Tissue levels of EtPhCbl

**Figure 5:**
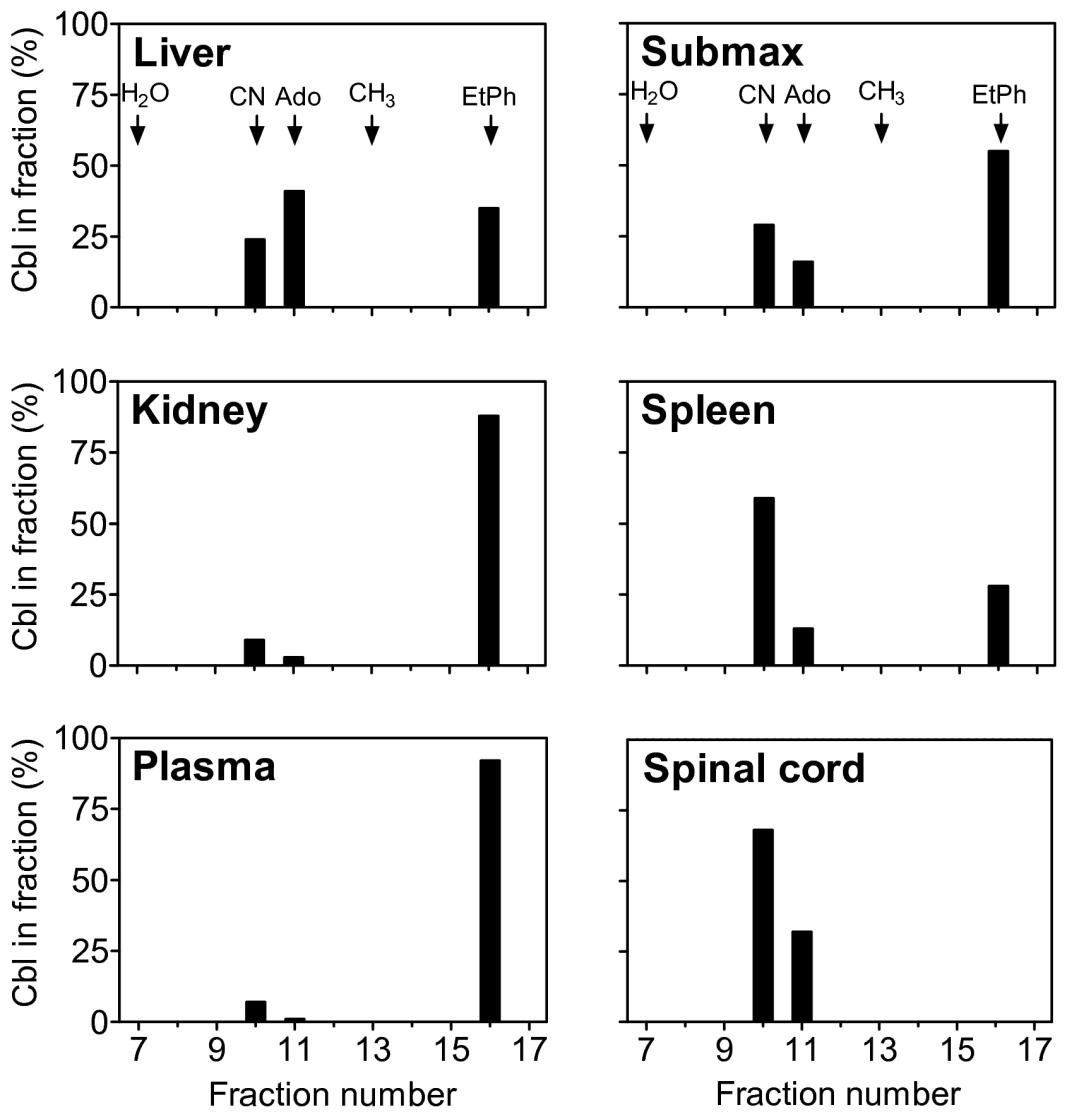
Forms of Cbl in pools of plasma or tissues of mice treated for four weeks with 3.5 nmol/day Co_β_-4-ethylphenyl-cob(III) alamin (EtPhCbl). Extracted Cbl was subjected to separation by HPLC. X-axis indicates the fraction number. Y-axis indicates the amount of Cbl present in each fraction expressed in percent of the total amount of Cbl recovered. The elution of reference preparations of each form of Cbl is indicated by the arrows. H_2_OCbl: aquocobalamin; CNCbl: vitamin B_12_; AdoCbl: 5′-deoxyadenosyl-cobalamin; CH _3_Cbl: methylcobalamin; EtPhCbl: Co_β_-4-ethylphenyl-cob(III) alamin.

To examine if EtPhCbl was able to enter the cells, we analyzed the molecular form of Cbl in pools of plasma and organ extracts from animals treated with EtPhCbl (Figure 5). We observed Cbl forms eluting similarly with CNCbl, AdoCbl and EtPhCbl, but no reactivity eluted like H_2_OCbl and CH _3_Cbl. This indicates that acid extraction in the presence of cyanide converts H_2_OCbl, and most likely also CH _3_Cbl, to CNCbl, while EtPhCbl and AdoCbl remain intact. As expected, EtPhCbl accounted for the major part of circulating Cbl in animals treated with EtPhCbl. In addition, EtPhCbl was present in all tissues examined except for the spinal cord. The EtPhCbl fraction of total Cbl was 90% in plasma and kidney, 55% in submaxillary gland, and around 30% in liver and spleen (Figure 5).

## Discussion

Recently, we described a new organometallic form of Cbl, EtPhCbl, which was inert against the action of the crucial enzyme CblC in *in vitro* enzyme assays, and therefore would not undergo a later conversion to the biological active AdoCbl and CH _3_Cbl [4]. Here, we report that mice treated with EtPhCbl for four weeks show signs of an impaired metabolism of Cbl characterized by tissue depletion of Cbl and an increased level of the metabolic markers of Cbl deficiency, MMA and tHCy.

The cellular uptake of Cbl is mediated by TC, and only forms of Cbl recognized by TC can enter the cells using this transport system. Under physiological conditions the TC transport system is far from saturated. This has two implications: Firstly treatment with CNCbl - as well as other forms of Cbl - results in tissue accumulation of the vitamin [7,11,12]; Secondly the TC transport system has sufficient capacity for transporting pharmacological Cbl derivatives into the cells without impairing the transport of endogenous Cbl. In accord with this, we recently reported tissue accumulation upon loading mice with cobinamide [7,12], an inactive form of Cbl recognized by mouse TC [5].

Since EtPhCbl binds to TC with an affinity comparable to that of H_2_OCbl and CNCbl (Figure 2), we expected to see tissue levels of EtPhCbl comparable to those observed when treating the animals with the same amounts of CNCbl. Unexpectedly, this was not the case. After four weeks of treatment with EtPhCbl, the total Cbl levels were high in plasma and low in the tissues compared to the mice treated with the same dose of CNCbl. Currently, we do not know whether the low level of tissue Cbl represents an impaired uptake or an increased export of EtPhCbl. Regardless of the mechanism, however, our observation has implications for the pharmacological use of the TC transport system. Several attempts have been made in order to use the TC mediated cellular uptake for carrying e.g. drugs into the cells [13,14]. Our results emphasize that the success of such an attempt depends not only on the binding of the Cbl derivative to TC, but also on the transport of the Cbl derivative into the cells and its ability to remain in the cells. Furthermore, this must occur without impairing the accumulation of endogenous Cbl. Our mouse model may prove very useful in exploring whether TC bound Cbl derivatives are accumulated in the tissues without impairing the accumulation of endogenous Cbl. Such studies are of obvious importance prior to introducing a new Cbl derivative in the clinical setting.

Interestingly, Cbl depletion upon treatment with EtPhCbl showed distinct differences between the various tissues studied. The most severe Cbl depletion was observed in the liver and the spleen, while the spinal cord was affected only to a limited degree (Figure 4). In addition, accumulation of EtPhCbl varied between the tissues. While almost all of the Cbl present in the kidney was EtPhCbl, nothing was observed in the spinal cord. Our results suggest tissue-specific differences related to the handling of Cbl. Such differences have previously been described for the kidney as compared to other tissues. The kidney has a dual Cbl uptake system. TC is recognized not only by its receptor, CD320, but, after filtration, also by the multifunctional receptor, megalin, present on the luminal membranes of the proximal tubules [15,16]. To our knowledge, little is known about differences in Cbl handling of the other organs studied. However, our results imply that the level and forms of Cbl in the spinal cord is left virtually unchanged despite severe alterations in other tissues of mice treated with EtPhCbl.

We initiated the treatment of mice with EtPhCbl to explore whether this would lead to a Cbl- deficient state. Our results show that the Cbl-deficient state is likely to be caused by a combination of Cbl depletion and tissue accumulation of EtPhCbl. At the end of the experiment, the mice treated with EtPhCbl showed an increase in MMA and tHcy of 3.4 and 1.5, respectively, when compared to control animals. The increase in MMA and tHcy is considerably higher than observed in other models of Cbl deficiency such as total gastrectomy [17], nutritional deprivation of Cbl [18] or treatment with cobinamide [7]. At the same time, the increase in MMA is much lower than in transgenic mice with a none-functioning Ado-Cbl dependent enzyme. MMA in the knockout mice is up to 500 µl/L at 8 weeks of age [19]. These observations suggest that our model creates a more profound state of Cbl deficiency than previously reported models. Simultaneously, our results indicate the presence of a considerable residual enzyme activity, even in animals where the total amount of Cbl in the liver is reduced to one third of that in the control animals and where more than half of the total Cbl present occurs as EtPhCbl.

In conclusion, we have shown that prolonged treatment of mice with the new Cbl derivative, EtPhCbl, induces a more severe Cbl deficiency than previous experimental models aimed at establishing Cbl deficiency. Surprisingly, Cbl deficiency was associated with cellular depletion of endogenous Cbl apparently caused by binding of EtPhCbl to TC at the expense of endogenous Cbl combined with an impaired cellular uptake of EtPhCbl. This observation points to the fact that binding of a Cbl derivative to TC does not necessarily predict cellular accumulation of the derivative. We suggest the use of animal models, such as ours, in order to explore the potential pharmacological benefits of new Cbl derivatives. A clinical useful derivative must enter the cells without impairing the transport of endogenous Cbl.
